# High Prevalence of Colistin-Resistant Encoding Genes Carriage among Patients and Healthy Residents in Vietnam

**DOI:** 10.3390/medicina60071025

**Published:** 2024-06-21

**Authors:** Viet Ha Le, Thi Diep Khong, Ngoc Quang Phan, Thi Hoa Tran, Hong Ngoc Vu, Dong Van Quyen, Van Thuan Hoang, Nam Thang Nguyen

**Affiliations:** 1 Thai Binh University of Medicine and Pharmacy, Thai Binh 410000, Vietnam; halv@tbmc.edu.vn (V.H.L.); diepkt@tbump.edu.vn (T.D.K.); quangpn@tbmc.edu.vn (N.Q.P.); hoatt@tbump.edu.vn (T.H.T.); ngocvh@tbump.edu.vn (H.N.V.); thuanhv@tbump.edu.vn (V.T.H.); 2 Biotechnology Department, Graduate University of Science and Technology, Vietnam Academy of Science and Technology, Hanoi 11307, Vietnam; dvquyen@ibt.ac.vn; 3 Institute of Biotechnology, Vietnam Academy of Science and Technology, Hanoi 11307, Vietnam

**Keywords:** colistin-resistant encoding genes, *mcr*, Vietnam

## Abstract

*Background and Objectives*: We aimed to investigate the carriage of colistin-resistant genes among both patients with a history of antibiotic exposure and apparently healthy adults with no recent healthcare contact. *Materials and Methods*: Stool swabs were collected from healthy people, and specimens were collected at the infection foci from the patients. Eleven primer/probe sets were used to perform the Multiplex Real-Time PCR assay with the QuantiNova Multiplex Probe PCR kit for screening the carriage of colistin-resistant genes (*mcr*-1 to *mcr*-10) and *16S rRNA* gene as internal control. *Results*: In total, 86 patients and 96 healthy residents were included. Twenty two patients (25.9%) were positive with at least one colistin-resistance encoding gene. The *mcr*-1 gene was the most frequent (16.5%), followed by *mcr*-9, *mcr*-6, and *mcr*-4 genes, where the prevalence was 11.8%, 10.6%, and 9.4%, respectively. No patient was positive with *mcr*-3, *mcr*-7, and *mcr*-8 genes. Eight patients (9.4%) were positive with multiple colistin-encoding genes. Twenty-three healthy people (24.0%) were positive with at least one colistin-resistance encoding gene, and the *mcr*-10 gene was the most frequent (27.0%), followed by the *mcr*-1, *mcr*-8, and *mcr*-9 genes, where the prevalence was 24.3%, 21.6%, and 13.5%, respectively. No person was positive with the *mcr*-2 and *mcr*-5 genes. *Conclusions*: Our findings underscore the urgent need for enhanced surveillance, infection control measures, and stewardship interventions to mitigate the spread of colistin resistance in Vietnam.

## 1. Introduction

The emergence and rapid dissemination of antibiotic resistance represents a formidable challenge to global public health, jeopardizing the efficacy of antimicrobial therapies and significantly complicating the management of infectious diseases [1]. This escalating crisis is fueled by several factors, including the overuse and misuse of antibiotics in human and veterinary medicine, as well as the widespread dissemination of resistant bacteria within healthcare facilities and the broader community. The resulting diminished effectiveness of available treatment options not only prolongs illness and increases the risk of severe complications, but also raises the specter of a post-antibiotic era where common infections could once again become life-threatening [2].

Among the diverse arsenal of antibiotics, colistin has garnered attention as a critical last-resort treatment for infections caused by multidrug-resistant Gram-negative pathogens. These include formidable adversaries such as *Acinetobacter baumannii*, notorious for its ability to survive in hospital environments and cause severe healthcare-associated infections, and *Pseudomonas aeruginosa*, a versatile opportunistic pathogen capable of colonizing diverse niches within the host and displaying intrinsic resistance to many antibiotics. Additionally, various members of the *Enterobacteriaceae* family, such as *Escherichia coli* and *Klebsiella pneumoniae*, have emerged as significant threats due to their ability to acquire and disseminate resistance determinants, leading to the emergence of carbapenem-resistant and, more recently, colistin-resistant strains [3].

Despite its historical use dating back several decades, colistin regained prominence as a treatment option due to the rise of multidrug-resistant bacteria that had exhausted other therapeutic avenues. However, the clinical utility of colistin is now severely compromised by the alarming increase in the prevalence of colistin-resistant strains worldwide [4]. This resurgence of resistance is predominantly attributed to the acquisition of mobile genetic elements carrying colistin resistance determinants, notably the *mcr* (mobilized colistin resistance) genes. These plasmid-mediated mechanisms confer resistance to colistin by modifying the bacterial cell membrane, thereby reducing the drug’s ability to disrupt membrane integrity and exert its bactericidal effect. The rapid dissemination of *mcr* genes among diverse bacterial species, facilitated by horizontal gene transfer and global travel, has fueled concerns about the imminent loss of this critical antibiotic as a therapeutic option [5].

Understanding the dynamics of colistin resistance is paramount for the development of effective strategies aimed at mitigating its profound impact on public health. While considerable research efforts have historically concentrated on clinical settings and healthcare-associated infections, there is a burgeoning recognition of the crucial importance of examining the carriage of colistin-resistant genes within the broader community, particularly among ostensibly healthy individuals [6]. This shift in focus stems from a growing awareness of the potential role played by asymptomatic carriers of antibiotic-resistant bacteria in fueling the spread of resistance and exacerbating the global challenge of antimicrobial resistance (AMR).

Healthy carriers of antibiotic-resistant bacteria represent a significant reservoir for the dissemination of resistance genes, acting as unwitting vectors capable of transmitting these genetic determinants to susceptible individuals and environmental reservoirs [7]. Their role in the transmission dynamics of AMR cannot be overstated, as they serve as a silent conduit for the propagation of resistant strains across diverse ecological niches, including households, community settings, and healthcare facilities. Furthermore, the interplay between human carriers and environmental reservoirs, such as water sources and agricultural settings, further amplifies the potential for the dissemination and persistence of resistance genes in the environment [8].

By examining the prevalence and determinants of colistin resistance in healthy populations, researchers can gain invaluable insights into the underlying ecological factors driving the emergence and perpetuation of resistance [9]. Such investigations may uncover nuanced interactions between human activities, environmental factors, and microbial ecology, shedding light on the complex interplay that governs the selection and dissemination of resistant bacteria [10]. Moreover, understanding the epidemiology of colistin resistance in healthy individuals can inform targeted interventions aimed at curbing the transmission of resistant strains and preserving the efficacy of colistin as a last-resort antibiotic [11].

This study aims to investigate the carriage of colistin-resistant genes among both patients with a history of antibiotic exposure and apparently healthy adults with no recent healthcare contact. The results of our work contribute to elucidating the extent of community transmission and in identifying potential sources and drivers of resistance dissemination. Such knowledge is essential for developing targeted interventions to limit the spread of colistin resistance and to preserve the efficacy of this critical antibiotic.

## 2. Materials and Methods

### 2.1. Participants and Study Design

This was a cross-sectional descriptive study conducted among patients hospitalized at Thai Binh general hospital from 26 October 2022 to 12 June 2023, and healthy residents living in Tien Hai district, Dong Hung district, and Thai Binh city of Thai Binh province during the period from October 2022 to September 2023.

### 2.2. Specimen Collection

Stool swabs were collected from healthy people and stored in a tube with 1 mL Cary-Blair transport medium (MELAB diagnostics, Hanoi, Vietnam), kept at 4 °C, and transported to the laboratory in the same day. The stool samples were analyzed within 24 h after being collected.

For patients with infectious diseases, specimen collected at the infection foci was divided in two parts: one for culture, isolation, identification, and antibiogram of the bacteria as routine, and the other for DNA extraction and detection of colistin resistance genes. Only patients who were positive with Gram-negative bacteria were included in the study.

### 2.3. Extract DNA from Healthy Human Stool Samples and Patient Samples

The tube containing the stool sample was shaken thoroughly so that the stool was dissolved in the solution. The solution was transferred into a new tube and centrifuged at 10.000 rpm for 1 min. Discard the supernatant and retain about 200 µL of residue for DNA extraction using the Qiagen Fast DNA Stool mini kit (Qiagen, Hilden, Germany) according to the manufacturer’s instructions. Most patient samples extracted DNA using the Qiagen DNA mini kit (Qiagen, Hilden, Germany), except for one stool sample, which was extracted using the Qiagen Fast DNA Stool mini kit according to the manufacturer’s instructions. All of the extracted DNA samples were stored at −30 °C until they were used for detection of colistin resistance genes with a Multiplex Real-time PCR.

### 2.4. Primers and Probes Design for Multiplex Real-Time PCR

Reference sequences of all known *mcr* variants were downloaded from the National Center for Biotechnology Information (NCBI) Reference Gene Catalogue (accessed on 23 June 2022) for designing primers and probes for a Multiplex Real-Time PCR assay. To increase the specificity of each primer/probe set, reference sequences of variants with high similarity (*mcr*-1, *mcr*-2, and *mcr*-6; *mcr*-3, *mcr*-7, *mcr*-9, and *mcr*-10) were grouped together and aligned using Clustal Omega. Aligned sequences were analyzed using BioEdit version 7.0 (Informer Technologies, Inc., Los Angeles, CA, USA) and FastPCR version 6.8 (PrimerDigital, Helsinki, Finland) softwares to design primer/probe set for each *mcr* variant so that each primer/probe set was unique to only one *mcr* variant. In addition, a primer/probe set was used to detect the *16S rRNA* gene of bacteria for internal control. The information on the primer/probe sets used in the study is presented in Supplementary Table S1. All of the primers and probes were purchased from Integrated DNA Technologies (Coralville, IA, USA).

### 2.5. Positive Control

DNA sequences of colistin resistance genes from *mcr*-1 to *mcr*-10 were cloned into pJET 1.2 vector using a CloneJET™ PCR Cloning Kit (ThermoFisherScientific, Waltham, MA, USA). Recombinant plasmids were transformed into DH10B-competent cells and cultured in LB broth at 37 °C overnight. Cultured samples were centrifuged to harvest bacterial cells, extracted of their recombinant plasmid using GeneJET Plasmid Miniprep Kit (Thermo Fisher Scientific, Waltham, MA, USA), and used as positive control.

### 2.6. Detection of Bacteria and Antibiotic-Resistant Encoding Genes

To detect colistin-resistant genes in healthy human stool samples and patient samples, we used 11 primer/probe sets to perform the Multiplex Real-Time PCR assay (Supplementary Table S1) with the QuantiNova Multiplex Probe PCR kit (Qiagen, Hilden, Germany). For each sample, we conducted 3 Multiplex Real-Time PCR reactions. The first reaction detected *mcr*-1, *mcr*-3, *mcr*-5, and *mcr*-10 genes. The second reaction detected *mcr*-4, *mcr*-6, and *mcr*-8 genes. The third reaction detected *mcr*-2, *mcr*-7, *mcr*-9, and *16S rRNA* genes. The volume of each reaction was 20 µL, including 5 µL 4× QuantiNova Multiplex PCR Master Mix, 1 µL 20× primer-probe mix (8 µM forward primer + 8 µM reverse primer + 5 µM probe for each *mcr* gene), 1 µL DNA template, and 13 µL Rnase-free water. The Multiplex Real-Time PCR assay was performed on a LightCycler 480 II system (Roche, Basel, Switzerland) with the following thermal program: 2 min for activation of DNA polymerase, 40 cycles with a denaturation step at 95 °C for 5 s, and an annealing/extension step at 60 °C for 30 s. Fluorescence data were collected at the end of the annealing/extension step. Each batch reaction is always performed with at least a positive and a negative control sample. The result of the Multiplex Real-Time PCR assay was analyzed using LightCycler 1.52 software, and samples with CT ≤ 35 were considered positive.

Because the study objective was to screen for colistin-encoding resistance genes, only specimens positive for at least one *mcr* gene were cultured on CHROMagar™ COL-APSE agar (CHROMagar™, Paris, France) to isolate colistin-resistant Gram-negative bacilli. Bacterial identification and antimicrobial susceptibility testing was performed on a Vitek-2 system (BioMérieux, Marcy-l’Étoile, France).

### 2.7. Statistical Analysis

The data have been exported in Excel form. Statistical analyses were carried out using STATA version 17.0 (StataCorp, College Station, TX, USA). Categorical variables were presented as percentages. Quantitative variables were presented as mean and standard deviation.

## 3. Results

### 3.1. Characteristics of Included Population

A total of 85 patients and 96 healthy residents were included, with the mean age being 65.0 ± 15.6 (range = 24–92) and 54.2 ± 15.2 (range = 18 to 84), respectively. A proportion of 44.7% (38/85) of patients and 33.3% (32/96) of healthy people were male (Table 1).

For the patients, 57.6% (49/85) of cases were diagnosed as urinary tract infection, which may or may not be accompanied by other diseases, so the samples collected were urine; 27.1% (23/85) of cases were diagnosed as localized infection (pus or fluid at the site of infection samples); 5.9% (5/85) of cases were diagnosed as respiratory tract infection (phlegm or respiratory tract fluid samples); 8.2% (7/85) of case were diagnosed as choledocholithiasis (bile fluid samples); and 1.2% (1/85) of cases was diagnosed as intestinal infection (stool sample) (Table 2).

### 3.2. Identification of Bacteria and Colistin-Resistance Encoding Gene

Among 85 patients with infectious diseases, 22 (25.9%) were positive with at least one colistin-resistance encoding gene. *The mcr*-1 gene was the most frequent (16.5%), followed by the *mcr*-9, *mcr*-6, and *mcr*-4 genes, where the prevalence was 11.8%, 10.6%, and 9.4%, respectively. No patient was positive with *mcr*-3, *mcr*-7, and *mcr*-8 genes. Eight patients (9.4%) were positive with multiple colistin-encoding genes (Table 3).

*Escherichia coli* was the most frequent bacteria identified (12/22, 54.5%), followed by *Klebsiella pneumoniae* (2/22, 9.1%), *Pseudomonas aeruginosa* (2/22, 9.1%), and *Citrobacter freundii* (2/22, 9.1%) (Supplementary Table S2).

Among 96 healthy persons, 23 (24.0%) were positive with at least one colistin-resistance encoding gene. *The mcr*-10 gene was the most frequent (27.0%), followed by the *mcr*-1, *mcr*-8, and *mcr*-9 genes, where the prevalence was 24.3%, 21.6%, and 13.5%, respectively. No person was positive with the *mcr*-2 and *mcr*-5 genes (Table 3).

In 23 patients who were positive with at least one colistin-resistance encoding gene, bacteria culture was negative in 6 patients (26.1%). One patient was positive with *E. coli* and *K. pneumoniae*. *Escherichia coli* was the most frequent bacteria identified (9/23, 39.1%), followed by *Klebsiella pneumoniae* (8/23, 34.8%). Only one patient (4.3%) was positive with *Proteus mirabilis* (Supplementary Table S3).

## 4. Discussion

The emergence and spread of colistin resistance pose significant challenges to global public health, particularly in regions where the therapeutic options for multidrug-resistant bacterial infections are limited [11]. In this cross-sectional descriptive study conducted in Vietnam, we investigated the prevalence of colistin-resistant encoding genes among both hospitalized patients and healthy residents.

Our findings reveal a concerning prevalence of colistin-resistant genes among both patient and healthy populations in Vietnam. Among patients with infectious diseases, 25.9% were positive for at least one colistin-resistance encoding gene. This suggests that colistin resistance is not only present, but that it is also actively circulating within healthcare settings, potentially complicating the management of infectious diseases and limiting treatment options [12,13].

Interestingly, we observed variations in the prevalence of specific colistin-resistant genes between patient and healthy populations. The *mcr*-1 gene was the most frequently detected among patients, whereas *mcr*-10 predominated among healthy individuals [13,14]. This disparity in gene prevalence between the two groups may reflect differences in exposure to selective pressure, such as antibiotic usage or environmental factors [15,16].

Furthermore, the detection of multiple colistin resistance genes in a subset of patients underscores the complexity of antimicrobial resistance mechanisms and the potential for the co-selection of resistance traits [17]. The co-existence of multiple resistance genes within individual bacterial isolates highlights the need for comprehensive surveillance and monitoring efforts to track the emergence and dissemination of resistance determinants [18].

The high prevalence of colistin resistance genes among healthy residents is particularly alarming, as it suggests that resistance may be circulating within the community, independent of healthcare-associated exposures [19,20]. This raises concerns regarding the potential for zoonotic transmission or environmental reservoirs of resistant bacteria, further highlighting the need for integrated One Health approaches to combat antimicrobial resistance [21].

Our study has several limitations that warrant consideration. Firstly, the cross-sectional nature of the study limits our ability to establish causal relationships or determine the temporal trends in colistin resistance. Additionally, the relatively small sample size and limited geographic scope may not fully capture the diversity of resistance patterns across different regions of Vietnam.

## 5. Conclusions

In conclusion, our findings underscore the urgent need for enhanced surveillance, infection control measures, and stewardship interventions to mitigate the spread of colistin resistance in Vietnam. Addressing the multifaceted drivers of resistance will require a coordinated and multidisciplinary approach involving healthcare providers, policymakers, veterinarians, environmental scientists, and the broader community.

## Figures and Tables

**Table 1:** Age and gender of the study population.

	Patient (*n* = 85)	Healthy Resident (*n* = 96)
Age	65.0 ± 15.6 (24–92)	54.2 ± 15.2 (18–84)
Gender	Male: 44.7% (38) Female: 55.3% (47)	Male: 33.3% (32) Female: 66.7% (64)

**Table 2:** Diagnosis and sample type of the patients and healthy residents.

	Sample Type	*n*	%
Patients (*n* = 85)			
Urinary tract infection	Urine	49	57.6
Localized infection	Pus or fluid at the site of infection	23	27.1
Respiratory tract infection	Sputum or respiratory tract fluid	5	5.9
Choledocholithiasis	Bile fluid	7	8.2
Intestinal infection	Feces	1	1.2
Healthy residents (*n* = 96)	Feces	96	100.0

**Table 3:** Distribution of colistin-resistance encoding genes among the studied population.

Colistin-Resistance Encoding Genes	Patients with Infectious Diseases		Healthy Persons	
	*n*	%	*n*	%
*mcr-1*	14	16.5	9	9.4
*mcr-2*	5	5.9	0	0
*mcr-3*	0	0	2	2.1
*mcr-4*	8	9.4	1	1. 0
*mcr-5*	2	2.4	0	0
*mcr-6*	9	10.6	1	1.0
*mcr-7*	0	0	1	1.0
*mcr-8*	0	0	8	8.3
*mcr-9*	10	11.8	5	5.2
*mcr-10*	1	1.2	10	10.4

## Data Availability

The original contributions presented in the study are included in the article and Supplementary Material, further inquiries can be directed to the corresponding author [N.T.N.].

## Supplementary Materials

The following supporting information can be downloaded at: https://www.mdpi.com/article/10.3390/medicina60071025/s1. Table S1: Primers and probes used in the study; Table S2: Detection of colistin resistant genes from patients’ samples and bacterial identification and antibiograms of Gram nagative bacilli isolated from patients’ samples; Table S3: Detection of colistin resistant genes from stool samples of healthy residents and bacterial identification and antibiograms of Gram nagative bacilli isolated from stool samples.
