# Supplementary material for: High Prevalence of Colistin-Resistant Encoding Genes Carriage among Patients and Healthy Residents in Vietnam
Source: Medicina (Kaunas). 2024 Jun 21;60(7):1025. doi: 10.3390/medicina60071025 (PMC11278595; doi:10.3390/medicina60071025)
Supplement: Supplementary file 1 [file medicina-60-01025-s001.zip › medicina-3054754-supplementary.pdf]

Table S1. Primers and probes used in the study.

Target gene	Primer name	Sequence (5'-3')	Product length (bp)
mcr-1	MCR1:F_340-365	CAAGCCGAGACCAAGGATCTATTAAA	157
	MCR1:R_476-496	CAAGACTTGCCACGATCAAGC	
	MCR1:RP_451-475	CYAN500-CCAATCGGCGCATCAAACCCTTGCC-BHQ1	
mcr-2	MCR2:F_7-29	TCACAKCACTCTTGGTATCGCTA	157
	MCR2:R_140-163	CCGCCATTGAGAYGATAAAGCCTA	
	MCR2:RP_106-134	FAM-TCCGATACAGGATAGACCGCCATCGCTTT-BHQ2	
mcr-3	MCR3:F_541-563	AACAATTCAAACCTCCAGCGTGA	161
	MCR3:R_677-701	AGRAACATCAACGTGGGCTTACTTT	
	MCR3:RP_645-673	HEX-TAGTATCCCGTTTTGCATCATCACCTAAA-BHQ2	
mcr-4	MCR4:F_663-682	GAATGCCAGTCGTAACCCGA	145
	MCR4:R_786-807	GTTAAACGCAATCAGCCCCTGA	
	MCR4:RP_709-737	CY5-CTCATTGAGCGCGCAGTTTCACCCACAAC-BHQ2	
mcr-5	MCR5:F_1392-1413	CGAGAAAGGCCTGTATCTCCAT	106
	MCR5:R_1476-1497	TTGGTCGGCATAAACCTGACTC	
	MCR5:FP_1433-1458	FAM-CGCCGGATGAGCAGATCAAGGTGCCG-BHQ2	
mcr-6	MCR6:F_33-57	GGTCAATCCCTATCTGTTGATGAGC	135
	MCR6:R_148-167	ACAAGCGCCGTTGAGATCAC	
	MCR6:RP_118-145	HEX-AGCCTGCGTTTTGTGCCATCGGATAAGT-BHQ1	
mcr-7	MCR7:F_416-436	GCTATCCGGCAAACTGGTACA	149
	MCR7:R_544-564	CACTATCTGCTTGCCCAGGAT	
	MCR7:FP_446-471	CY5-CCATCAGGGCTGGTGCTCTGGCCTTC-BHQ2	
mcr-8	MCR8:F_663-686	GGAGGATGCTGTACGTCCAATATA	115
	MCR8:R_757-777	GGTTACCCGCGAATAGCCATT	
	MCR8:RP_718-741	FAM-TGCTCTGGCGGTTTCGCCCACGAC-BHQ2	
mcr-9	MCR9:F_535-559	GGCCGCAATAACTCGACATTGAATA	114
	MCR9:R_627-648	CAGCGTCTGGAAAGGCACTTTA	
	MCR9:FP_566-595	HEX-TCATCCCGGCGAACTACGCTTACAGCACTT-BHQ2	
mcr-10	MCR10:F_91-114	CATTTCTACGATATCCTGAGCCGT	110
	MCR10:R_177-200	ACCGAGAAGGGCATAAACACAAAG	
	MCR10:RP_119-148	CY5-TGGAGATGACGAACCCCGCCCTCACATGTT-BHQ2	
16S rRNA	16S:F_883-901	GGAGTACGRCCGCAAGGTT	88
	16S:R_949-970	TCGCGTTGCWTCGAATTAAACC	
	16S:RP_924-948	CYAN500-ACATGCTCCACCGCTTGTGCGGGCC-BHQ1	

Note: Underlined letters are degenerate base: R = A or G; Y = C or T; K = G or T; W = A or T.

Table S2. Detection of colistin resistant genes from patients' samples and bacterial identification and antibiograms of Gram nagative bacilli isolated from patients' samples.

ID	Specimen	Multiplex Real-time PCR results										Identification	Antibiograms													
		mcr-1	*mcr*-2	*mcr*-3	*mcr*-4	*mcr*-5	*mcr*-6	*mcr*-7	*mcr*-8	*mcr*-9	*mcr*-10		AMP	CTX	CAZ	FOX	MEM	STR	KAN	GEN	CIP	NAL	TET	CHL	SXT	FOF
P1	Sputum									+		*Pseudomonas aeruginosa*	R	R	S	R	S	R	R	S	S	R	S	R	R	S
P2	Pus	+										*Citrobacter freundii*	R	R	R	R	I	R	S	S	R	I	R	I	R	S
P3	Pus						+					*Escherichia coli*	R	S	S	R	S	R	S	S	S	S	R	R	S	S
P4	Urine									+		*Sphingomonas paucimobilis*	R	S	S	R	S	I	S	S	S	S	S	R	S	S
P5	Urine										+	*Escherichia coli*	R	S	S	S	S	R	S	S	R	S	S	S	R	S
P6	Pus	+										*Escherichia coli*	S	R	S	S	S	S	S	S	S	S	S	S	S	S
P7	Urine						+					*Klebsiella oxytoca*	I	S	I	S	S	R	I	S	R	R	R	S	R	S
P8	Bile fluid									+		*Escherichia coli*	R	R	R	R	S	R	R	S	R	R	R	R	R	S
P9	Pus									+		*Escherichia coli*	R	S	S	S	S	R	S	S	R	R	R	R	R	S
P10	Urine				+		+					*Raoultella ornithinolytica*	R	R	I	R	S	S	S	S	I	S	S	S	S	S
P11	Urine	+	+		+		+					*Proteus mirabilis*	R	S	S	S	I	R	R	S	I	R	R	R	R	S
P12	Urine	+			+		+			+		*Escherichia coli*	R	R	R	I	I	R	S	S	R	R	R	S	R	S
P13	Urine	+			+					+		*Escherichia coli*	R	R	S	S	S	R	I	R	R	R	R	S	R	S
P14	Urine	+	+		+	+	+			+		*Pseudomonas aeruginosa*	R	R	S	R	S	R	R	R	S	R	R	R	I	S
P15	Urine	+	+		+	+	+			+		*Escherichia coli*	R	R	R	S	S	I	I	R	R	R	S	S	R	S
P16	Pus	+	+		+		+			+		*Escherichia coli*	R	R	R	R	S	R	I	R	R	R	R	R	R	S
P17	Pus	+	+		+		+			+		*Escherichia coli*	R	R	R	R	S	R	R	R	R	R	R	R	R	S
P18	Urine	+										*Escherichia coli*	R	S	S	S	S	R	R	R	S	S	R	R	R	S
P19	Urine	+										*Escherichia coli*	R	R	R	S	S	S	S	S	S	R	R	S	S	S
P20	Pus	+										*Klebsiella pneumoniae*	R	S	S	R	S	R	S	S	I	S	R	S	S	S
P21	Pus	+										*Klebsiella pneumoniae*	R	S	S	R	S	S	S	S	I	I	I	S	S	S
P22	Urine	+										*Citrobacter freundii*	R	I	I	R	S	S	R	S	R	R	S	S	R	S

Antibiotic abbreviation: AMP: ampicillin; CTX: cefotaxime; CAZ: ceftazidime; FOX: cefoxitin; MEM: meropenem; STR: streptomycin; KAN: kanamycin; GEN: gentamycin; CIP: ciprofloxacin; NAL: nalidixic acid; TET: tetracycline; CHL: chloramphenicol; SXT: sulfamethoxazole/trimethoprim; FOF: fosfomycin.

Table S3. Detection of colistin resistant genes from stool samples of healthy residents and bacterial identification and antibiograms of Gram nagative bacilli isolated from stool samples.

Sample ID	Identification	Multiplex Real-time PCR results										Antibiograms													
		mcr-1	*mcr*-2	*mcr*-3	*mcr*-4	*mcr*-5	*mcr*-6	*mcr*-7	*mcr*-8	*mcr*-9	*mcr*-10	AMP	CTX	CAZ	FOX	MEM	STR	KAN	GEN	CIP	NAL	TET	CHL	SXT	FOF
H46	*Escherichia coli*	+										R	S	S	R	S	R	R	R	I	S	R	R	R	S
	Klebsiella pneumoniae											R	S	S	S	S	S	S	S	S	S	S	S	S	S
H49	*Proteus mirabilis*						+	+	+			R	R	S	S	S	R	S	S	R	R	R	R	R	I
H51	*Escherichia coli*	+										R	I	S	S	S	I	R	S	R	S	R	R	R	S
H52	*Klebsiella pneumoniae*										+	R	S	S	S	S	R	R	R	R	R	R	R	R	S
H53	*Escherichia coli*	+		+								S	R	S	S	S	R	S	S	S	S	R	R	R	S
H54	*Klebsiella pneumoniae*		+									S	R	R	R	R	R	R	R	R	R	R	S	S	S
H55	*Escherichia coli*	+										R	I	S	S	S	S	S	S	S	S	S	S	S	S
H57	*Negative*				+																				
H70	*Escherichia coli*	+										R	S	S	S	S	R	I	S	S	S	R	R	R	S
H75	*Negative*									+															
H79	*Escherichia coli*	+										R	S	S	S	S	R	I	S	R	I	R	S	R	S
H80	*Negative*								+	+	+														
H81	*Klebsiella pneumoniae*										+	R	R	I	S	S	R	S	S	R	S	R	S	R	S
H82	*Klebsiella pneumoniae*								+	+	+	R	S	S	S	S	S	S	S	R	I	R	R	S	S
H83	*Klebsiella pneumoniae*										+	R	S	S	R	S	S	S	S	S	S	S	S	S	S
H86	*Klebsiella pneumoniae*										+	R	S	S	I	S	I	S	S	S	S	S	S	S	S
H87	*Negative*										+														
H89	*Escherichia coli*	+							+	+	+	R	R	I	S	S	S	R	R	R	R	R	R	R	S
H92	*Negative*								+																
H93	*Negative*								+		+														
H94	*Klebsiella pneumoniae*								+			R	S	S	S	S	S	S	S	S	S	S	S	S	S
H99	*Escherichia coli*	+										R	S	S	S	S	I	S	S	I	S	I	R	R	S
H105	*Escherichia coli*	+									+	R	S	S	S	S	R	S	R	I	R	R	R	R	S

Antibiotic abbreviation: AMP: ampicillin; CTX: cefotaxime; CAZ: ceftazidime; FOX: cefoxitin; MEM: meropenem; STR: streptomycin; KAN: kanamycin; GEN: gentamycin; CIP: ciprofloxacin; NAL: nalidixic acid; TET: tetracycline; CHL: chloramphenicol; SXT: sulfamethoxazole/trimethoprim; FOF: fosfomycin.
